# A 2-month intervention study of preventive clothing against mosquito bites among malnourished and well-nourished children under 5 years of age living on the Atlantic Ocean Coast of Lagos, Southwest Nigeria

**DOI:** 10.1186/s12936-020-3143-x

**Published:** 2020-02-05

**Authors:** Bamgboye M. Afolabi, Titilola M. Afolabi, Abiodun Ogunwale, Adewunmi Aiyesetenikan

**Affiliations:** 1 Health, Environment and Development Foundation, Lagos, Nigeria; 2 Midwestern University, Glendale, AZ USA; 3 Project Hope, Washington, DC USA

**Keywords:** Malnutrition, Stunting, Wasting, Malaria, Hypoglycemia, Anemia, Mosquito Armor^®^, Atlantic Ocean Coast, Nigeria

## Abstract

**Background:**

Malnutrition is appreciated as a global leading paediatric burden that indirectly or directly contributes to child mortality. In children, malnutrition has profound effects on health and development; and has been associated with poor outcomes in paediatric diseases. However, it is not clear if malnourished children are at an increased risk of having malaria. This study was conducted to evaluate the risk of malaria infection in children with malnutrition.

**Methods:**

The study design was pre-post. A protective clothing against mosquitoes (pCAM) was given to 102 under-five children in two coastal communities, after screening for malaria parasitaemia. The children’s weight, height and body temperature were measured at recruitment. Blood was also taken at recruitment and monthly for malaria parasitaemia, haemoglobin concentration and random blood sugar. The parents/care-givers were visited daily for 1 month only, after recruitment, to ensure that the children wore the pCAM daily from 5 pm and the children were followed up monthly for 2 months.

**Results:**

Of the 102 study participants, 60 (24 males and 36 females) were rapid diagnostic test (RDT)-positive at recruitment, indicating 58.8% prevalence of malaria parasitaemia. The prevalence of malnutrition and of stunting were 32.3% (33/102) and 54.9% (56/102), respectively, while 7.8% (8/108) children were wasted. Twenty (60.6%) of the malnourished children and 30 (53.6%) of those stunted were RDT-positive at recruitment. At the first post-intervention screening, only 7 (31.8%) of the malnourished and 13 (28.9%) of those stunted were RDT-positive. Malnourished and stunted children were 2.57 times and 2.31 times more likely to be malaria infected (OR = 2.57, 95% CI 0.97, 6.79; OR = 2.31, 95% CI 1.01, 5.26 respectively). Malnourished females were 2.72 times more likely to be RDT-positive compared to malnourished males (OR = 2.72, 95% CI 0.54, 11.61) and stunted females were 1.73 times more likely to the positive for malaria parasites than stunted males (OR 1.73, 95% CI 0.59, 5.03). The prevalence of anaemia at recruitment decreased from 82.4 to 69.6% after intervention. The mean haemoglobin concentration (g/dl) at recruitment was significantly lower (P < 0.05) than that at 1st and 2nd post-intervention measurements (9.6 ± 1.4, t = − 3.17, P-value = 0.0009 and 10.2 ± 1.3, t = − 2.64, P-value = 0.004, respectively). Mean random blood sugar (mg/dl) of females (91.8 ± 12.7) was significantly lower (t = 2.83, P-value = 0.003) than that of males (98.5 ± 11.2).

**Conclusion:**

Results from this study suggest a higher risk of malaria infection among malnourished and lower risks among stunted and wasted children. Females were at a higher risk of malnutrition, stunting and wasting than males. Protective clothing against malaria seemed to reduce malaria infection and improve anaemia status.

## Background

Malnutrition and malaria are two deleterious diseases that significantly affect children in sub-Saharan Africa (SSA). In Nigeria, available data from the 2001–2003 Food Consumption and Nutrition Survey suggests that 42% of children under five were stunted, 25% underweight, and 9% wasted [1]. Nigeria also has the world’s heaviest malaria burden, with about 51 million cases and 207,000 deaths reported annually (approximately 30% of the total malaria burden in Africa), while 97% of the total population (approximately 173 million) is at risk of infection [2]. The disease accounts for 60% of outpatient visits to hospitals, approximately 11% maternal mortality and 30% child mortality, especially among children less than 5 years [3, 4]. Malaria and malnutrition co-exist in sub-Saharan Africa and when combined can result in severe morbidity and mortality, however, the association between the two diseases is controversial.

The major malaria vectors in Nigeria are *Anopheles gambiae* sensu lato and *Anopheles funestus*. *Anopheles melas* is also found in lower density on the Atlantic Ocean coast of the country. The *Anopheles* species are known to bite between 2200 and 0500 h in most parts of malaria-endemic Africa, justifying the recommendation that people, including children and pregnant women sleep under long-lasting insecticide-treated mosquito nets (LLINs), towards achieving malaria control [5–7].

An earlier study in the same study site however showed that mosquitoes biting could start as early as 1900 h when children and mothers/caregivers are still outdoor [8]. The probability of early mosquito bite times prompted the production and use of protective clothing during the early hours of the evening when mosquitoes are just coming out to feed and children are not yet in bed. According to the World Health Organization (WHO), clothing can offer protection from biting insects and that the treatment of clothing with an insecticide or repellent can deter insects from biting through clothes [9].

The co-morbidities associated with malaria are extensive and include anemia. Malaria is the foremost source of anaemia where the disease is endemic. Anaemia, measured by a reduced concentration of functional haemoglobin (Hb) in the blood, poses serious health concerns in children as it is associated with impaired mental and physical development [10]. It is also recognized as a major cause of morbidity and mortality among under-fives, especially in Africa [11]. Other associated contributors to anaemia are other infectious diseases and nutritional deficiencies [12].

Although controversial, studies suggest that there is a likely association between the risk of malaria and malnutrition in children, however, there is a lack of evidence as to whether preventing malaria in children is associated with improved nutrition. Malnourishment can be assessed using anthropometry, a widely used as a tool to estimate the nutritional status of populations and to monitor the growth and health of individuals. The three most frequently used anthropometric indices are weight-for-height, height-for-age, and weight-for-age. The anthropometric evaluation of a community supports identification of groups at risk of poor functional outcomes (morbidity and mortality) in dire need of further appraisal or intervention [13]. Additionally, glucose is an instant source of energy and the preferred essential substrate for cerebral energy metabolism, can be utilized as an immediate marker of under nutrition in children [14].

There are very few studies that report a combination of malaria, anaemia, nutritional anthropometry and serum glycaemia levels especially in communities living on the Atlantic Ocean coastline in Nigeria. Data are also very scarce on the use of pCAM in sub-Saharan Africa. This study may, therefore, be the first to survey malaria, anaemia and hypoglycaemia with a backdrop of nutritional anthropometry among children under the age of 5 years. The slow progress observed in reducing malaria morbidity and mortality among the at-risk groups is probably because, at dusk, children and pregnant women have already been bitten by infected mosquitoes before sleeping under Long-lasting insecticide-treated nets (LLINs). Public health interventions, in the form of protective clothing against mosquitoes ought to, theoretically, slow or diminish the current prevalence of malaria and simultaneously lessen the downstream expenditure connected with it. Should a solution to the heavy burden of malaria exists, it probably lies in the basic improvement in population health that can be achieved by protective clothing against the vector that causes the disease, as a supplement to already existing malaria commodities. Since LLINs protect people when they are in bed, an appropriate, safe and affordable device is necessary to protect them while they are mobile and expose to the risk of mosquito bites. There is a dearth of knowledge on the availability and efficacy of specific protective clothing against mosquitoes and it is vital to close this gap. The overall aim of this study was to contribute to further reduction in the incidence of malaria, especially among children under the age of five, thus lowering its morbidity and mortality in endemic African Region. The main objective was to evaluate whether pCAM reduces the risk of malaria infection in children. Secondary objectives were (i) to assess various levels of malnutrition, stunting and wasting in general and among rapid diagnostic test (RDT)-positive and RDT-negative children; (ii) to evaluate prevalence of anaemia in general and among RDT-positive and RDT-negative children; (iii) to determine the prevalence of hypoglycaemia relative to nutritional status and to malaria status.

## Methods

**Fig. 1:**
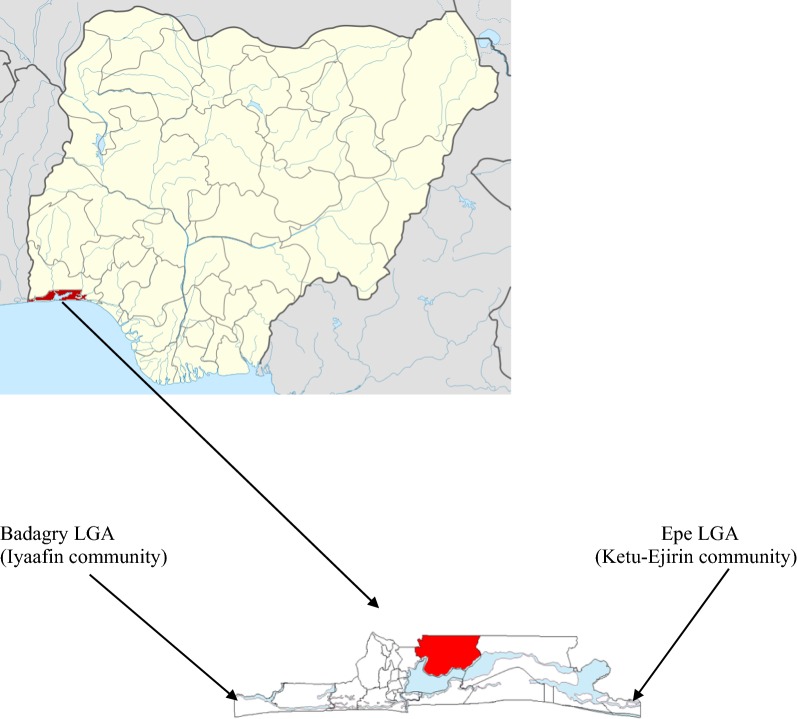
Composite map of Lagos State in Nigeria showing study sites—Iyaafin Community (Badagry Local Government Area) and Ejirin Community (Epe Local Government Area) of Lagos State

The protocol for this study was approved by the National Health Research Ethics Committee, Lagos State Branch (No. NHREC 04/04/2008 Ref. No. RFC/10/06/367). The study design was longitudinal and cross-sectional. It was also an intrusion study in that an intervention—pCAM—was provided. In this regard, it was also a prophylactic trial aimed at preventing malaria. The study took place at Iyaafin, a community of about 3000 people in Badagry Local Government Area (pop. 241,093) which lies at 6° 25′ N 2° 53′ E and at Ejirin, with a population of approximately 4000 people, located in Epe Local Government Area (pop. 181,734) lying at 6° 35′ N 3° 59′ E (Fig. 1). While most of the adult population at Iyaafin were mostly agrarian farmers and traders, the major occupation of Epe inhabitants was fishing. This study was conducted during the first 4 months of the year during which agricultural produce was expected to be in surplus and available for participants. A simple random sampling technique, using sample fraction, was used to identify 150 households where children below the age of 5 years and older siblings resided. If an identified household did not have a child below the age of five, the immediate next habitable house was chosen as replacement. Informed consent was obtained verbally from each child’s caretaker before enrollment in the study. Community consent for the study also was obtained from the Traditional Head of each community of study. Children were eligible for the study if they had no evidence of severe acute malaria, congenital diseases, other chronic illnesses, and have been living in the communities for at least 1 year. Participants were excluded from the study if they were visitors to the communities, admitted to the hospital, or if parents/caregivers did not give consent to partake in the study.

Study participants underwent anthropometry conducted by trained, experienced fieldworkers. Weight was measured to the nearest 5 g using a Salter digital weighing scale. Recumbent length (for children 2 years old or below) or standing height (for those above 2 years) was measured to the nearest 0.1 cm using a locally-made rigid height board with tape rule. Weight-for-age Z-scores (WAZ), height-for-age Z-scores (HAZ) and weight-for-height Z-scores (WHZ) were calculated using WHO Anthro Software v3.2.2. Vitals were also recorded with body temperature measured using digital thermometer. Venous blood samples were aseptically obtained to measure hemoglobin concentration for anaemia status and random blood sugar (RBS) for hypoglycaemia, and for malaria infection using rapid diagnostic test (RDT). Malaria infection was assessed using RDT kits (Carestart™ Access Bio Inc., USA), which tests for the *Plasmodium falciparum* histidine rich protein-2 (PfHRP-2), a water-soluble parasite-specific protein released from parasite-infected erythrocytes. PfHRP-2 is produced throughout the parasite’s 48-h life cycle, with approximately 90% released at the moment of schizont rupture [15]. Haemoglobin concentration was evaluated using Hemocue portable photometer as stated by the manufacturer and random blood sugar (RBS) was measured using commercially available Glucometer kit. For haemoglobin estimation, RDT and RBS, a small drop of blood, obtained with minimal pain by aseptically pricking the pulp of the thumb or the heel using spring loaded lancing device containing Unilet disposable lancets and Autolet platforms (Owen Mumford, Oxford, England), was placed on a disposable test strip that the Hemocue, RDT kit or the Glucometer read after a few minutes. A line across the strip (RDT positive) sensitive for *P. falciparum* indicated recent infection within weeks, while Glucometer and Hemocue displayed the blood glucose level in unit of mg/dl and the haemoglobin concentration in unit of g/dl, respectively. All variables of interest were measured once every month for 2 months. The first measurement served as baseline information before the intervention. Thereafter, five field workers visited the parents/caregivers at least four times per week, in the 1st month, to ensure the study children wore the pCAM every day, after 1700 h. The first post-intervention measurements were taken between 30 and 31 days after commencement of study. In the 2nd month of study, i.e. 31–60 days, social workers deliberately did not visit parents/caregivers to determine variations in study parameters when the parents/caregivers were visited to ensure child wore the protective clothing compared to when not visited. The second intervention measurements took place after absence of social workers. There was no crossover or washout period in the study.

### Sample size calculation

At the initial stage, a pilot study, using RDT, was implemented to calculate the point prevalence of malaria among children in Badagry and in Epe, the results of which were 56.1% and 42.3%, respectively. These figures were used to calculate the sample size for each community of study according to the formula of Altman described by Whitley and Ball [16]:

$${\text{n}} = \frac{{p1\left( {1 - p1} \right) + p2\left( {1 - p2} \right)}}{{\left( {p1 - p2} \right) ^{2} }} . C_{{{\text{p}}1\;{\text{power}}}}$$

where n represents the sample to be determined, and p1 and p2 the prevalence of malaria among children in Badagry and in Epe, respectively. The power of the study, set at 0.9, and probability (1 − β) of rejecting null hypothesis when it is false, was taken as 0.9. At a significance level (α) of 0.05, Cp1 power corresponds to 10.51. Thus,

$${\text{n}} = \frac{{\left[ {\left( {0.56(1 - 0.56} \right) + 0.42\left( {1 - 0.42} \right)} \right]}}{{\left( {0.56 - 0.42} \right) ^{2} }} \times 10. 5 1 {\text{ or 263 in each community}} .$$

Attrition was discountenanced in the study and the total sample size for each community was estimated at 250.

### Training of field workers and social workers

Nine field workers were expected to be part of the study team consisting of two nurses, three people to serve questionnaires, three to coordinate laboratory equipment and one for field logistics. A 2-day training session was conducted to familiarize all fieldworkers and laboratory scientists not only with the technical aspect of the survey, but also with inter-personal relationship, communication line, dispute resolution and logistics. The training involved going through each step of the survey—understanding why each question in the questionnaire was asked and the order of the questions, appropriate timing to most likely encounter parents/caregivers at home, mode of dressing to the field and according respect to every respondent. The laboratory scientists were trained regarding patient flow, prompt response to participants, safety and keeping samples secured. Three Social Workers from the Local Government were recruited and re-trained in conducting home visit, note-taking during visit, basic health education and de-briefing.

### The intervention

**Fig. 2:**
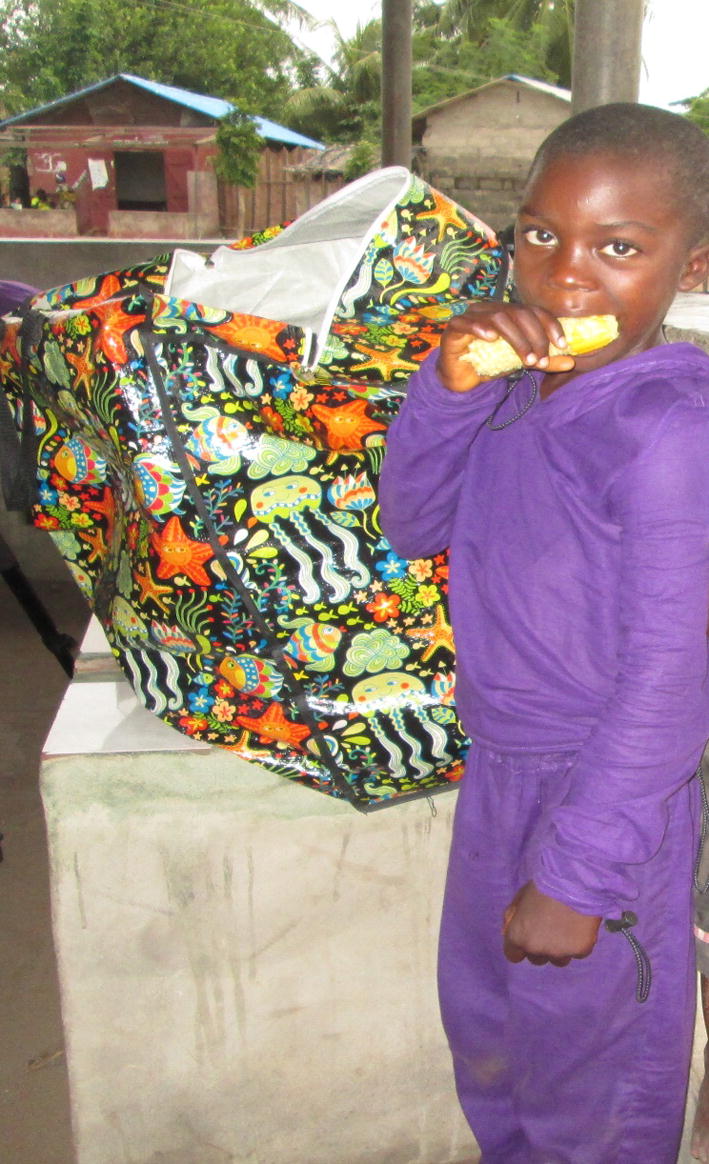
A child wearing “Moskeeto Armor^®^” during the study. The protective clothing is made to cover the head, arms and feet while awake or sleeping

A pCAM (Moskeeto Armor^®^, manufactured and donated by ING Activewear^®^, USA), is an insecticide-treated protective clothing against malaria. Its fabrics tested in laboratory contained 1.26 mg/m^2^ of permethrin. The body measurement and shape of each child was taken by a professional tailor and the pCAM was sewn, as a jacket with hood and trouser, according to each child’s measurement, to cover the entire body exposing only the hand and feet (Fig. 2). Each under-five in the study was given a sewn pCAM to wear daily, which could be washed when dirty and worn again. In addition, the research team made some effort to give parents/caregivers health education on breast-feeding, child immunization and nutrition whenever the parents/caregivers bring their children for screening.

### Definitions

For the purpose of this study RDT-positive meant that the child had a recent falciparum infection where if RDT-negative, the child did not have such infection. Classical definition of malnutrition was assumed as a child with WAZ < − 2; of stunting as a child with HAZ < − 2 and of wasting (thinness) as a child with WHZ < − 2 [17]. Anaemia was taken to be Hb of < 11 g/dl, mild anaemia as Hb of 10–10.9 g/dl, moderate anaemia as Hb of 7.0–9.9 g/dl and severe anaemia as Hb of < 7 g/dl [18]. There is no specific hypoglycaemia cut-off point for under-fives in sub-Saharan Africa. Thus, this community-based study assumes a threshold value of 90 mg/dl and below for low random blood sugar (LRBS) though, in clinical practice, a value of < 3.9 mmol/l (70 mg/dl) is used because of the potential for the glucose to fall further [19].

### Data entry and statistical analysis

Primary data were manually entered into field log-book, later transferred to an electronic system for data security and finally reviewed by the field supervisor. When detected, an error, omission or inappropriate data entry necessitated field workers/laboratory scientists returning to the mother/caregiver to correct the data. Data analysis was performed using Stata statistical software (version 13; StataCorp, Texas USA). Non-parametric tests were used for determining the significance of associations of variables. There was no missing data. Analysis carried out included frequency of proportions, bivariate (cross-tabulation), and multivariate regression analysis, adjusting for possible confounders. Continuous variables were compared between groups by Mann–Whitney U test, and proportions were compared by χ^2^ test. Odds ratios (ORs) and 95% CIs were calculated. Results were expressed as mean (± standard deviation [SD]) and presented as Tables and Figures. Level of significance was taken as P < 0.05.

## Results

### General characteristics of study children (Table 1)

A total of 108 children were enrolled in the study, of which six children were lost to follow-up due to travels (n = 3), parental hospitalization (n = 1), child illness (n = 1) and withdrawal of consent (n = 1) leaving 45 (44.1%) males and 57 (55.9%) females.

**Table 1:** Demographic and clinical characteristics of study children at baseline relative to gender

Variable	Total (n = 102, 100.0%)					Male (n = 45, 44.1%)					Female (n = 57, 55.9%)				
	Mean (± sd)	Min.–max.	Percentile			Mean (± sd)	Min.–max.	Percentile			Mean (± sd)	Min.–max.	Percentile		
			25%	Median	75%			25%	Median	75%			25%	Median	75%
Age (m)	34.8 (15.2)	4.76–59.9	23.0	36.6	48.0	32.7 (16.6)	4.8–59.9	19.2	31.5	44.0	36.5 (14.0)	7.4–59.7	27.1	37.3	48.5
	t-test (P-value)					− 1.23 (0.11)									
Weight (kg)	12.1 (3.0)	5.0–20.1	10.0	11.0	15.0	12.0 (2.9)	7.0–20.0	10.0	11.0	14.0	12.1 (3.2)	5.0–20.1	10.0	12.0	15.0
	t-test (P-value)					− 0.17 (0.43)									
Height (cm)	85.6 (12.0)	62.5–115.6	77.0	86.0	94.0	84.3 (12.4)	55.0–112.0	75.5	85.0	94.9	86.5 (11.8)	67.5–115.6	80.0	86.0	94.0
	t-test (P-value)					− 0.91 (0.18)									
Temp. °C	36.6 (0.3)	36.0–38.6	36.5	36.6	36.7	36.6 (0.3)	36.1–37.6	36.5	36.6	36.7	36.6 (0.4)	36.0–38.6	36.5	36.6	36.7
	t-test (P-value)					0.00 (1.00)									
Hemoglobin (g/dl)	9.6 (1.4)	5.4–12.8	8.5	9.6	10.5	9.4 (1.3)	6.3–12.8	8.5	9.3	10.1	9.7 (1.5)	5.4–12.1	8.8	9.8	10.8
	t-test (P-value)					− 1.08 (0.14)									
Random blood sugar	94.8 (12.5)	64.0–119.0	89.0	96.5	102.0	98.5 (11.2)	72.0–115.0	94.0	98.0	107.0	91.8 (12.7)	64.0–119.0	81.0	94.0	100.0
	t-test (P-value)					2.83 (0.003)									
WAZ-score	− 1.31 (1.32)	− 3.99 to 2.22	− 2.39	− 1.26	− 0.42	− 1.25 (1.28)	− 3.86 to 1.34	− 2.39	− 1.33	− 0.42	− 1.36 (1.35)	− 3.99 to 2.22	− 2.39	− 1.23	− 0.48
	t-test (P-value)					0.42 (0.34)									
HAZ-score	− 1.98 (1.51)	− 5.09 to 2.19	− 3.13	− 2.16	− 0.86	− 2.10 (1.44)	− 4.3 to 1.05	− 3.34	− 2.12	− 0.84	− 1.89 (1.57)	− 5.09 to 2.19	− 2.83	− 2.16	− 1.06
	t-test (P-value)					− 0.52 (0.30)									
WHZ-score	− 0.14 (1.50)	− 3.97 to 3.19	− 1.18	− 0.04	0.82	− 0.05 (1.52)	− 3.4 to 3.19	− 1.26	− 1.95	0.87	− 0.21 (1.44)	− 3.97 to 2.85	− 1.16	− 0.01	0.81
	t-test (P-value)					− 0.70 (0.24)									
RDT +ve	Freq. (%)		60 (58.8)			24 (53.3)					36 (63.2)				
RDT −ve	Freq. (%)		42 (41.2)			21 (46.7)					21 (36.8)				
χ^2^ (P-value)						1.00 (0.32)									
OR (95% CI)						1.50 (0.68, 3.32)									
Dermatological findings															
Tinea															
Capitis	Freq. (%)		24 (23.5%)			17 (37.8)					7 (12.3)				
Corporis	Freq. (%)		16 (157%)			10 (22.2)					6 (10.5)				
χ^2^ (P-value)						0.30 (0.59)									
OR (95% CI)						1.46 (0.38, 5.57)									

Overall clinical characteristics of study subjects indicated that the mean haemoglobin concentration (g/dl) was 9.6 (1.4), with no statistical difference between males (9.4 ± 1.3) and females (9.7 ± 1.5). However, the mean random blood sugar (RBS) of males (98.5 ± 11.2) was significantly higher (t = 2.83, P-value = 0.003) than that of females (91.8 ± 12.7). The overall means (± sd) of weight-for-age Z-score, height-for-age Z-score and weight-for-height Z-score were − 1.31 (1.32), − 1.99 (1.52) and − 0.13 (1.47), respectively, with no noteworthy alterations between the two sexes.

### Fever

Mean axillary temperature of the children was 36.6 ± 0.3 °C. Almost all the children (100, 98.0%) presented at recruitment with axillary temperature of < 37.5 °C. The only 2 (2.0%) children (1 male and 1 female) with axillary temperature ≥ 37.5 °C were RDT-negative but were still given appropriate anti-malarial recommended by the Federal Ministry of Health.

### Dermatological diseases and allergy

Among the 33 malnourished (WAZ < − 2) children 9 (27.3%) had *tinea capitis* (ringworm) among whom 7 (77.8%) were males and 2 (22.2%) were females. Only 6 (1.2%) of the malnourished children (3 males and 3 females) had *tinea corporis*. Male children were approximately 1½ times more likely to have *tinea capitis* than female children (χ^2^ = 0.30, P-value = 0.59, OR = 1.46, 95% CI 0.38, 5.57). Those with *tinea capitis* or *tinea corporis* were given Whitfield ointment to apply at the affected parts of the body. There were no other dermatological diseases after the study.

### Rapid diagnostic tests for malaria

In all, 60 (58.8%) of the children were RDT-positive and the rest (42, 41.2%) were RDT negative. Females were 1.5 times more likely to be RDT positive than males (χ^2^ = 1.00, p-value = 0.32, OR = 1.50, 95% CI 0.68, 3.32).

### Nutritional anthropometry

The anthropometric indices of the study children is as shown in Table 2. The overall mean (± sd) weight-for-age Z-score (WAZ) was − 1.31 (1.3), with males recording mean WAZ of − 1.24 (1.3) and females − 1.36 (1.4). The proportion of malnourished (WAZ ≤ − 2) female children (20/57, 35.1%) was higher than that of males (13/45, 28.9%) though the difference did not approach any level of significance. Consequently, females were 1.33 times more likely to be malnourished compared to their male counterparts (χ^2^ = 0.44, P-value = 0.51, OR = 1.33, 95% CI 0.57, 3.09). Approximately 56% of female children were stunted with HAZ score of ≤ − 2 compared to about 53% of the male children. Females were 3.38 time more likely to be stunted than males (χ^2^ = 8.46, P-value = 0.003, OR = 3.38, 95% CI 1.47, 7.78). Females were also 1.35 as likely to be more wasted than males (χ^2^ = 0.15, P-value = 0.69, OR = 1.35, 95% CI 0.30, 5.96).

**Table 2:** Anthropometric indices of study children at baseline

Variable	Sub variable	< 60 months old												t	P-value	χ^2^	P-value	OR	95% CI
		All				Male				Female									
		n = 102				n = 45 (44.1%)				n = 57 (55.9%)									
		Mean	± sd	Freq.	%	Mean	± sd	Freq.	%	Mean	± sd	Freq.	%						
WAZ-score	All	− 1.31	1.3	102	100.0	− 1.24	1.30	45	44.1	− 1.36	1.4	57	55.9	0.44	0.33	–			
	< − 2.0	− 2.83	0.6	33	32.3	− 2.82	0.5	13	28.9	− 2.83	0.6	20	35.1	0.05	0.48	0.44	0.51	1.33	0.57, 3.09
	− 2.0 to − 2.0	− 0.61	0.8	68	66.7	− 0.57	0.8	32	71.1	− 0.65	0.8	36	63.2	0.41	0.34	0.70	0.40	0.70	0.30, 1.61
	> 2.0	2.22	0.0	1	1.0	0.0	0.0	0	0.0	2.22	0.0	1	1.7	–	–	0.00	1.00	Undefined	Undefined
HAZ-score	All	− 1.99	1.5	102	100.0	− 2.13	1.4	45	43.0	− 1.89	1.6	57	57.0	0.80	0.21	–			
	< − 2	− 3.07	0.8	56	54.9	− 3.21	0.7	24	53.3	− 2.97	0.8	32	56.1	− 1.19	0.12	8.46	0.003	3.38	1.47, 7.78
	− 2.0 to − 2.0	− 0.71	0.9	45	44.1	− 0.82	0.8	21	46.7	− 0.63	1.0	24	42.1	− 0.71	0.24	0.21	0.65	0.83	0.37, 1.83
	> 2.0	2.19	0.0	1	1.0	0.0	0.0	0	0.0	2.19	0.0	1	1.8	–	–	0.00	1.00	Undefined	Undefined
WHZ-score	All	− 0.13	1.5	102	100.0	− 0.02	1.5	45	43.0	− 0.21	1.4	57	57.0	0.65	0.26	–			
	≤ − 2	− 3.07	0.7	8	7.8	− 2.84	0.6	3	6.7	− 3.20	0.7	5	8.8	0.77	0.23	0.15	0.69	1.35	0.30, 5.96
	− 2.0 to − 2.0	− 0.14	1.0	84	82.4	− 0.23	1.0	35	77.8	− 0.07	1.0	49	86.0	− 0.72	0.24	1.15	0.28	1.75	0.63, 4.88
	> 2.0	2.33	0.4	10	9.8	2.29	0.5	7	15.5	2.42	0.4	3	5.2	− 0.44	0.34	2.98	0.08	0.30	0.07, 1.24

### RDT status and nutritional anthropometry (Table 3)

#### Pre-intervention screening

At pre-intervention screening, 33 (32.4%) children were malnourished (WAZ < − 2) of whom 20 (60.6%) were RDT-positive and 13 (39.4%) were RDT-negative. Of the 68 (66.6%) that were well-nourished, 40 (58.8%) were RDT-positive and 28 (41.2%) were RDT-negative. Prior to intervention, malaria-positive children were approximately 1.2 times more likely to be malnourished than to be well-nourished (χ^2^ = 1.40, P-value = 0.24, OR = 0.62, 95% CI 0.28, 1.37) but only 0.62 as likely to be stunted (HAZ < − 2) than not to be stunted (χ^2^ = 1.40, P-value = 0.24, OR = 0.62, 95% CI 0.28, 1.37).

**Table 3:** Pre- and post-screening anthropometric indices of RDT-positive and RDT-negative under-five children wearing pCAM

Variable	Pre-intervention screening												
	Weight-for-age Z-score (WAZ)					Height-for-age Z-score (HAZ)				Weight-for-height Z-score (WHZ)			
	Total		< − 2	− 2 to +2	> + 2	Total	< − 2	− 2 to + 2	> + 2	Total	< − 2	− 2 to +2	> + 2
	n	102	33	68	1	102	56	45	1	102	8	84	10
	%	100.0	32.4	66.6	1.0	100.0	54.9	44.1	1.0	100.0	7.8	82.4	9.8
All													
RDT +ve	Freq.	60	20*	40*	0	60	30**	29	1	60	2	54	4
	%	58.8	60.6	58.8	0.0	58.8	53.6	64.4	100.0	58.8	25.0	64.3	40.0
RDT −ve	Freq.	42	13	28	1	42	26	16	0	42	6	30	6
	%	41.2	39.4	41.2	100.0	41.2	46.4	35.6	0.0	41.2	75.0	35.7	60.0
Risk ratio (95% confidence interval)			1.05 (0.59, 1.87)				0.82 (0.58, 1.16)				0.21 (0.05, 1.00)		

1st post-intervention screening													
	n	102	22	80	0	102	45	55	2	102	11	86	5
	%	100.0	21.6	78.4	0.0	100.0	44.1	53.9	2.0	100.0	10.8	84.3	4.9
All													
RDT +ve	Freq.	43	7*	36*	0	43	13**	30**	0	43	4	37	2
	%	42.2	31.8	45.0	0.0	42.2	28.9	54.5	0.0	42.2	36.4	43.0	40.0
RDT −ve	Freq.	59	15	44	0	59	32	25	2	59	7	49	3
	%	57.8	68.2	55.0	0.0	57.8	71.1	45.5	100.0	57.8	63.6	57.0	60.0
Risk ratio (95% confidence interval)			0.64 (0.29, 1.43)				0.54 (0.32, 0.90)				0.78 (0.24, 2.49)		

2nd post-intervention screening													
	n	102	19	83	0	102	40	59	3	102	12	88	2
	%	100.0	18.6	81.4	0.0	100.0	39.2	57.8	2.9	100.0	11.8	86.3	2.0
All													
RDT +ve	Freq.	40	7*	33	0	40	14	23	3	40	7	33	0
	%	39.2	36.8	39.8	0.0	39.2	35.0	39.0	100.0	39.2	58.3	37.5	0.0
RDT −ve	Freq.	62	12	50	0	62	26	36	0	62	5	55	2
	%	60.8	63.2	60.2	0.0	60.8	65.0	61.0	0.0	60.8	41.7	62.5	100.0
Risk ratio (95% confidence interval)			0.90 (0.39, 2.10)				0.90 (0.54, 1.50)				2.10 (0.72, 6.16)		

*χ^2^ = 3.73, P-value = 0.05, OR = 2.57, 95% CI 0.97, 6.79

**χ^2^ = 3.99, P-value = 0.04, OR = 2.31, 95% CI 1.01, 5.26

#### At first post-intervention screening, after wearing pCAM and parents/caregivers were given instruction to ensure children wear pCAM

The proportion of RDT-positive children dropped from 60 (58.8%) at recruitment, to 43 (42.2%) at 1st post-intervention screening and finally to 40 (39.2%) at the 2nd post-intervention screening. This drop was most pronounced among malnourished children of who 60.6% (20/33) were RDT positive at recruitment, 31.8% (7/22) at 1st post-intervention measurement. Nevertheless, the proportion of RDT-positive children increased to 36.8% (7/19) at 2nd post-intervention measurement, when mothers/caregivers were not visited and prompted to ensure children wear the pCAM.

There was a marginally significant reduction (χ^2^ = 3.73, P-value = 0.05, OR = 2.57, 95% CI 0.97, 6.79) in the proportion of malnourished children that were malaria-positive at pre-intervention screening (20, 60.6%), compared to those that were malaria-positive at 1st post-intervention screening (7, 31.8%) after wearing pCAM. The odds of malnourished children testing positive for malaria was lower (0.57) at first post-intervention screening (χ^2^ = 1.22, P-value = 0.27, OR = 0.57, 95% CI 0.21, 1.55) compared to pre-intervention screening. Similarly, there was a significant reduction (χ^2^ = 3.99, P-value = 0.04, OR = 2.31, 95% CI 1.01, 5.26) in the proportion of stunted children that were malaria-positive at 1st post-intervention screening (13, 28.9%) when compared to those (30, 53.6%) at pre-intervention screening. Prior to intervention, the risks of malaria among malnourished (WAZ < − 2) and stunted (HAZ < − 2) children were 1.05 (95% CI 0.59, 1.87) and 0.82 (95% CI 0.58, 1.16) respectively. At first post-intervention screening, after wearing pCAM, the risks were reduced to 0.64 (95% CI 0.29, 1.43) and 0.54 (95% CI 0.32, 0.90), respectively.

#### At second post-intervention screening, after wearing pCAM and parents/caregivers were not visited to give instruction to ensure children wear pCAM

At the second post-intervention screen, after which the parents/caregivers were not visited to be informed of ensuring their children wear the pCAM by 1700 h, the proportion of malnourished RDT-positive children increased to 36.8% (7/19) and that of stunted RDT-positive children also increased to 35.0%. The risk of malnourished and of stunted children to be RDT-positive also increased to 0.90 (95% confidence interval 0.39, 2.10) and 0.90 (95% confidence interval 0.54, 1.50) respectively.

### Gender, RDT and nutritional anthropometry (Table 4)

Females were approximately 1.4 times more likely to be malnourished (χ^2^ = 0.52, P-value = 0.47, OR = 1.36, 95% CI 0.59, 3.18) and 1.1 times more likely to be stunted (χ^2^ = 0.08, P-value = 0.78, OR = 1.12, 95% CI 0.51, 2.46) than males. Malnourished females were 2.72 times more likely to be RDT-positive (χ^2^ = 1.88, P-value = 0.17, OR = 2.72, 95% CI 0.64, 11.61) than malnourished males. Stunted females were also 1.73 times more likely to be RDT-positive (χ^2^ = 0.99, P-value = 0.31, OR = 1.73, 95% CI 0.59, 5.03) than stunted males. At pre-intervention screening, 53.3% (24/45) of male children were RDT-positive compared to 63.2% (63/57) of the female children. However, at first post-intervention screening, 31.1% (14/45) males and 50.9% (29/57) females were RDT positive, and at second post-intervention screening the proportion of RDT-positive males and females increased to 37.8% (17/45) and 40.3% (23/57), respectively.

**Table 4:** Gender related anthropometric indices of RDT-positive and RDT-negative under-five children wearing pCAM

Variable	Stat.	Pre-intervention screening																							
		Males												Females											
		Weight-for-age Z-score				Height-for-age Z-score				Weight-for-height Z-score				Weight-for-age Z-score				Height-for-age Z-score				Weight-for-height Z-score			
		Total	< − 2	− 2 to +2	> + 2	Total	< − 2	− 2 to +2	> + 2	Total	< − 2	− 2 to +2	> + 2	Total	< − 2	− 2 to +2	> + 2	Total	< − 2	− 2 to +2	> + 2	Total	<− 2	− 2 to +2	> + 2
	n	45	13^!^	32	0	45	24^!!^	21	0	45	3	35	7	57	20^!^	36	1	57	32^!!^	24	1	57	5	49	3
	%	100.0	28.9	71.1	0.0	100.0	53.3	46.7	0.0	100.0	6.7	77.8	15.5	100.0	35.1	63.2	1.7	100.0	56.1	42.1	1.8	100.0	8.8	85.9	5.3
RDT +ve	Freq.	24	6*	18	0	24	11	13	0	24	0	21	3	36	14*	22	0	36	19	16	1	36	2	33	1
	%	53.3	38.5	56.2	0.0	53.3	45.8	61.9	0.0	53.3	0.0	60.0	42.9	63.2	70.0	61.1	0.0	63.2	59.4	66.7	100.0	63.2	40.0	67.3	33.3
RDT −ve	Freq.	21	7	14	0	21	13	8	0	21	3	14	4	21	6	14	1	21	13	8	0	21	3	16	2
	%	46.7	61.5	43.8	0.0	46.7	54.2	38.1	0.0	46.7	100.0	40.0	57.1	36.7	30.0	38.9	100.0	36.7	40.6	33.3	0.0	36.7	60.0	32.7	66.7

1st post-intervention measurement																									
	N	45	10	35	0	45	22	23	0	45	2	39	4	57	12	45	0	57	23	32	2	57	9	47	1
	%	100.0	22.2	77.8	0.0	100.0	48.9	51.1	0.0	100.0	4.4	86.7	8.9	100.0	21.1	78.9	0.0	100.0	40.4	56.1	3.5	100.0	15.8	82.5	1.8
RDT +ve	Freq.	14	3	11	0	14	5	0	0	14	0	13	1	29	4	0	0	29	8	21	0	29	4	24	1
	%	31.1	30.0	31.4	0.0	31.1	22.7	0.0	0.0	31.1	0.0	33.3	25.0	50.9	33.3	0.0	0.0	50.9	34.8	65.6	0.0	50.9	44.4	51.1	100.0
RDT −ve	Freq.	31	7	24	0	31	17	0	0	31	2	26	3	28	8	0	0	28	15	11	2	28	5	23	0
	%	68.9	70.0	68.6	0.0	68.9	77.3	0.0	0.0	68.9	100.0	66.7	75.0	49.1	66.7	0.0	0.0	49.1	65.2	34.6	100.0	49.1	55.6	48.9	0.0

2nd post intervention measurement																									
	N	45	7	38	0	45	21	23	1	45	3	40	2	57	12	45	0	57	19	36	2	57	9	48	0
	%	100.0	15.6	84.4	0	100.0	46.7	51.1	2.2	100.0	6.7	88.9	4.4	100.0	21.1	78.9	0.0	100.0	33.3	63.2	3.5	100.0	15.8	84.2	0.0
RDT +ve	Freq.	17	3	14	0.0	17	9	7	1	17	1	16	0	23	4	19	0	23	5	16	2	23	6	17	0
	%	37.8	42.9	36.8	0	37.8	42.9	30.4	100.0	37.8	33.3	40.0	0.0	40.3	33.3	42.2	0.0	40.3	26.3	44.4	100.0	40.3	66.7	35.4	0.0
RDT −ve	Freq.	28	4	24	0.0	28	12	16	0	28	2	24	2	34	8	26	0	34	14	20	0	34	3	31	0
	%	62.2	57.1	63.7	0	62.2	57.1	69.6	0.0	62.2	66.7	60.0	100.0	59.7	66.7	57.8	0.0	59.7	73.7	55.6	0.0	59.7	33.3	64.6	0.0

*χ^2^ = 1.88, P-value = 0.17, OR = 2.72, 95% CI 0.64, 11.61

**χ^2^ = 1.01, P-value = 0.31, OR = 1.73, 95% CI 0.59, 5.03

^!^χ^2^ = 0.52, P-value = 0.47, OR = 1.36, 95% CI 0.59, 3.18

^!!^χ^2^ = 1.88, P-value = 0.17, OR = 2.72, 95% CI 0.64, 11.61

### Clinical indices of RDT-positive and RDT-negative study children by gender (Table 5, Figs. 3, 4)

The mean *temperature* (°C) of the study children was not significantly varied at recruitment (36.6 ± 0.3), 1st post-intervention (36.6 ± 0.3) and 2nd post-intervention (36.7 ± 0.3) measurements. The overall mean *haemoglobin* (g/dl) at recruitment (9.6 ± 1.4) significantly increased at 1st post-intervention measurement (10.2 ± 1.3; t = − 3.17, P-value = 0.0009) and at 2nd post-intervention measurement (10.1 ± 1.3; t = 2.64, P-value = 0.03). The mean hemoglobin concentration of RDT-positive and RDT-negative children increased from 9.4 g/dl and 9.7 g/dl at recruitment to 10.0 g/dl and 10.2 g/dl, respectively in 1st and 2nd post-intervention measurements.

**Table 5:** Pre- and post-screening clinical indices of RDT-positive and RDT-negative under-five children wearing pCAM, relative to gender

Variable	Sub-variable	Total		All				Male				Female			
				RDT positive		RDT negative		RDT positive		RDT negative		RDT positive		RDT negative	
		Freq. (%)	Mean (± sd)	Freq. (%)	Mean (± sd)	Freq. (%)	Mean (± sd)	Freq. (%)	Mean (± sd)	Freq. (%)	Mean (± sd)	Freq. (%)	Mean (± sd)	Freq. (%)	Mean (± sd)
At recruitment															
Temp. (°C)	All	102 (100.0)	36.6 (0.3)	60 (58.8)	36.6 (0.2)	42 (41.2)	36.7 (0.4)	24 (53.5)	36.6 (0.2)	21 (46.5)	36.6 (0.3)	36 (63.2)	36.6 (0.2)	21 (36.8)	36.7 (0.5)
	< 37.5	100 (98.0)	36.6 (0.2)	60 (100.0)	36.6 (0.2)	40 (95.1)	36.6 (0.2)	24 (100.0)	36.6 (0.2)	20 (95.0)	36.6 (0.3)	36 (100.0)	36.6 (0.2)	20 (95.2)	36.6 (0.2)
	≥ 37.5	2 (2.0)	38.1 (0.7)	0 (0.0)	0 (0.0)	2 (4.9)	38.1 (0.7)	0 (0.0)	0 (0.0)	1 (5.0)	37.6 (0.0)	0 (0.0)	0 (0.0)	1 (4.8)	38.6 (0.0)
Hb (g/dl)	All	102 (100.0)	9.6 (1.4)	60 (58.8)	9.4 (1.54)	42 (41.2)	9.7 (1.3)	24 (53.3)	9.3 (1.4)	21 (46.7)	9.6 (1.1)	36 (63.2)	9.5 (1.5)	21 (36.8)	9.9 (1.5)
	≥ 11.0	18 (17.7)	11.5 (0.6)	11 (18.3)	11.5 (0.6)	7 (16.7)	11.6 (0.4)	4 (16.7)	11.5 (1.0)	2 (9.5)	11.6 (0.1)	7 (19.4)	11.5 (0.5)	5 (23.8)	11.6 (0.5)
	10.0–10.9	23 (22.5)	10.4 (0.3)	11 (18.3)	10.4 (0.4)	12 (28.6)	10.5 (0.3)	4 (16.7)	10.3 (0.4)	6 (28.6)	10.3 (0.3)	7 (19.4)	10.4 (0.3)	6 (28.6)	10.6 (0.3)
	7.0–9.9	57 (55.9)	8.8 (0.7)	35 (58.4)	8.8 (0.7)	22 (52.4)	8.9 (0.8)	15 (62.5)	8.7 (0.7)	13 (61.9)	8.9 (0.7)	20 (55.6)	8.9 (0.8)	9 (42.8)	8.9 (0.9)
	< 7.0	4 (3.9)	6.2 (0.6)	3 (5.0)	6.2 (0.8)	1 (2.4)	6.2 (0.0)	1 (4.1)	6.3 (0.0)	0 (0.0)	0 (0.0)	2 (5.6)	6.2 (1.1)	1 (4.8)	6.2 (0.0)
RBS (mg/dl)	All	102 (100.0)	94.8 (12.5)	60 (58.8)	95.0 (12.4)	42 (41.2)	94.4 (12.6)	24 (53.3)	98.6 (12.0)*	21 (46.7)	98.4 (10.6)*	36 (63.2)	92.6 (12.3)*	21 (36.8)*	90.4 (13.5)
	≥ 100	38 (37.3)	106.8 (5.5)	25 (41.7)	106.1 (4.9)	13 (31.0)	108.2 (6.5)	12 (52.2)	107.5 (5.2)	9 (45.0)	108.0 (5.9)	13 (36.1)	104.8 (4.3)	4 (19.0)	108.5 (8.7)
	90–99	36 (35.3)	94.8 (3.0)	20 (33.3)	94.3 (3.2)	16 (38.1)	95.3 (2.7)	9 (34.8)	95.1 (3.1)	8 (35.0)	94.9 (3.2)	11 (30.6)	93.6 (3.3)	8 (38.1)	95.8 (2.2)
	70–89	25 (24.5)	79.9 (5.6)	13 (21.7)	79.5 (5.3)	12 (28.6)	80.4 (6.1)	3 (13.0)	73.7 (1.5)	4 (20.0)	83.8 (5.7)	10 (27.8)	81.2 (4.7)	8 (38.1)	78.8 (5.9)
	< 70	3 (2.9)	66.0 (2.0)	2 (3.3)	65.0 (1.4)	1 (2.4)	68.0 (0.0)	0 (0.0)	0 (0.0)	0 (0.0)	0 (0.0)	2 (5.6)	65.0 (1.4)	1 (4.8)	68.0 (0.0)
Pearson’s R (P-value)				0.14 (0.13)		− 0.12 (0.24)		0.26 (0.048)		− 0.38 (0.02)		0.02 (0.89)		0.10 (0.52)	
1st post-intervention screening															
Temp. (°C)	All	102 (100.0)	36.6 (0.3)	43 (42.2)	36.6 (0.4)	59 (57.0)	36.5 (0.3)	14 (32.6)	36.6 (0.4)	31 (67.4)	36.5 (0.3)	29 (50.9)	36.6 (0.4)	28 (49.1)	36.6 (0.3)
	< 37.5	99 (97.1)	36.5 (0.2)	41 (95.3)	36.5 (0.2)	58 (98.3)	36.5 (0.3)	13 (92.9)	36.5 (0.2)	31 (100.0)	36.5 (0.3)	28 (96.6)	36.6 (0.1)	27 (96.4)	36.5 (0.3)
	≥ 37.5	3 (2.9)	37.9 (0.5)	2 (4.7)	38.1 (0.6)	1 (1.7)	37.6 (0.0)	1 (7.1)	37.6 (0.0)	0 (0.0)	0 (0.0)	1 (3.4)	38.5 (0.0)	1 (3.6)	37.6 (0.0)
Hb (g/dl)	All	102 (100.0)	10.2 (1.3)	43 (43.0)	9.9 (1.3)	59 (57.0)	10.4 (1.2)	14 (32.6)	9.5 (1.6)	31 (67.4)	10.3 (1.3)	29 (50.9)	10.0 (1.2)	28 (49.1)	10.4 (1.2)
	≥ 11.0	31 (30.4)	11.6 (0.6)	10 (23.3)	11.4 (0.4)	21 (35.6)	11.7 (0.6)	3 (21.4)	11.2 (0.1)	10 (32.3)	11.8 (0.7)	7 (24.2)	11.5 (0.5)	11 (39.3)	11.6 (0.6)
	10.0–10.9	25 (24.5)	0.4 (0.3)	13 (30.2)	10.4 (0.2)	12 (20.3)	10.4 (0.4)	4 (28.6)	10.5 (0.1)	7 (22.6)	10.4 (0.3)	9 (31.0)	10.4 (0.3)	5 (17.9)	10.5 (0.4)
	7.0–9.9	45 (44.1)	9.1 (0.8)	19 (44.2)	8.8 (0.9)	26 (44.1)	9.3 (0.7)	6 (42.9)	8.5 (1.0)	14 (45.1)	9.2 (0.8)	13 (44.8)	9.0 (0.9)	12 (42.8)	9.4 (0.7)
	< 7.0	1 (1.0)	6.6 (0.0)	1 (2.3)	6.6 (0.0)	0 (0.0)	0 (0.0)	1 (7.1)	6.6 (0.0)	0 (0.0)	0 (0.0)	0 (0.0)	0 (0.0)	0 (0.0)	0 (0.0)
RBS (mg/dl)	All	102 (100.0)	93.2 (15.2)	43 (43.0)	94.1 (18.6)	59 (57.0)	92.4 (12.4)	14 (32.6)	101.7 (13.1)	31 (67.4)	93.6 (12.2)	29 (50.9)	90.5 (19.9)	28 (49.1)	91.1 (12.6)
	≥ 100	34 (33.3)	108.1 (5.4)	17 (39.5)	109.4 (5.9)	17 (28.8)	106.7 (4.7)	8 (57.1)	111.5 (5.4)	10 (32.3)	106.4 (5.1)	9 (31.0)	107.4 (5.9)	7 (25.0)	107. (4.4)
	90–99	32 (31.4)	94.4 (2.9)	13 (30.2)	93.5 (2.7)	19 (32.2)	95.1 (3.0)	4 (28.6)	92.5 (3.1)	10 (32.3)	95.5 (2.8)	9 (31.0)	94.0 (2.5)	9 (32.1)	94.6 (3.2)
	70–89	31 (30.4)	81.6 (5.1)	10 (23.3)	83.1 (3.8)	21 (35.6)	80.9 (5.6)	2 (14.3)	81.0 (5.7)	10 (32.3)	81.9 (5.9)	8 (27.6)	83.6 (3.5)	11 (39.3)	80.0 (5.4)
	< 70	5 (4.9)	55.2 (24.8)	3 (7.0)	47.3 (31.6)	2 (3.4)	67.0 (2.8)	0 (0.0)	0 (0.0)	1 (3.1)	65.0 (0.0)	3 (10.3)	47.3 (31.6)	1 (3.6)	69.0 (0.0)
Pearson’s R (P-value)				− 0.09 (0.44)		0.06 (0.48)		0.18 (0.35)		0.14 (0.25)		0.08 (0.56)		− 0.03 (0.82)	
2nd post-intervention screening															
Temp. (°C)	All	102 (100.0)	36.7 (0.3)	40 (39.2)	36.7 (0.3)	62 (60.8)	36.6 (0.2)	17 (39.5)	36.6 (0.2)	28 (60.5)	36.7 (0.2)	23 (40.3)	36.8 (0.4)	34 (59.7)	36.6 (0.2)
	< 37.5	100 (98.0)	36.6 (0.2)	38 (95.0)	36.6 (0.2)	62 (100.0)	36.6 (0.2)	17 (100.0)	36.6 (0.2)	28 (100.0)	36.7 (0.2)	21 (91.3)	36.7 (0.2)	34 (100.0)	36.6 (0.2)
	≥ 37.5	2 (2.0)	37.9 (0.4)	2 (5.0)	37.9 (0.4)	0 (0.0)	0 (0.0)	0 (0.0)	0 (0.0)	0 (0.0)	0 (0.0)	2 (8.7)	37.9 (0.4)	0 (0.0)	0 (0.0)
Hb (g/dl)	All	102 (100.0)	10.1 (1.3)	40 (39.2)	10.0 (1.4)	62 (60.8)	10.2 (1.3)	17 (39.5)	10.2 (1.8)	28 (60.5)	9.9 (1.2)	23 (40.3)	9.8 (1.1)	34 (59.7)	10.4 (1.4)
	≥ 11.0	24 (23.5)	11.8 (0.7)	6 (15.0)	12.1 (0.9)	18 (29.0)	11.7 (0.6)	4 (23.5)	12.3 (1.0)	5 (19.2)	11.6 (0.6)	2 (8.7)	11.7 (0.6)	13 (38.2)	11.7 (0.7)
	10.0–10.9	33 (32.3)	10.5 (0.3)	16 (40.0)	10.4 (0.3)	17 (27.4)	10.5 (0.3)	7 (41.2)	10.5 (0.2)	7 (26.9)	10.6 (0.2)	9 (39.1)	10.3 (0.3)	10 (29.4)	10.4 (0.3)
	7.0–9.9	43 (42.2)	9.1 (0.7)	17 (42.5)	9.0 (1.0)	26 (42.0)	9.1 (0.6)	5 (29.4)	8.7 (1.3)	16 (53.9)	9.1 (0.6)	12 (52.2)	9.1 (0.8)	10 (29.4)	9.2 (0.5)
	< 7.0	2 (2.0)	6.5 (0.1)	1 (2.5)	6.5 (0.0)	1 (1.6)	6.4 (0.0)	1 (5.9)	6.5 (0.0)	0 (0.0)	0 (0.0)	0 (0.0)	0 (0.0)	1 (2.9)	6.4 (0.0)
RBS (mg/dl)	All	102 (100.0)	90.3 (12.8)	40 (39.2)	91.0 (14.4)	62 (60.8)	89.9 (11.8)	17 (39.5)	92.8 (16.0)	28 (60.5)	91.9 (11.9)	23 (40.3)	89.7 (13.2)	34 (59.7)	88.2 (11.6)
	≥100	23 (22.6)	106.8 (9.3)	10 (25.0)	107.7 (12.9)	13 (18.3	106.1 (5.8)	4 (23.5)	112.5 (20.3)	8 (23.1)	107.0 (6.7)	6 (26.1)	104.5 (4.5)	5 (14.7)	104.6 (4.3)
	90–99	31 (30.4)	94.6 (2.8)	13 (32.5)	94.8 (3.2)	18 (30.0)	94.6 (2.6)	6 (35.3)	93.7 (3.3)	7 (26.9)	93.3 (2.3)	7 (30.4)	95.7 (3.0)	11 (32.3)	95.4 (2.6)
	70–89	44 (43.1)	80.9 (5.5)	16 (40.0)	79.1 (5.8)	28 (46.7)	81.9 (5.2)	7 (41.2)	80.7 (6.4)	13 (50.0)	81.9 (5.3)	9 (39.1)	77.8 (5.3)	15 (44.1)	81.9 (5.3)
	< 70	4 (3.9)	66.0 (1.2)	1 (2.5)	65.0 (0.0)	3 (5.0)	66.3 (1.2)	0 (0.0)	0 (0.0)	0 (0.0)	0 (0.0)	1 (4.4)	65.0 (0.0)	3 (8.8)	66.3 (1.2)
Pearson’s R (P-value)				0.14 (0.24)		0.11 (0.26)		0.16 (0.24)		0.15 (0.39)		0.17 (0.20)		0.07 (0.63)	

*Pearson’s correlation coefficient (r) = − 0.268, P-value = 0.006

**Fig. 3:**
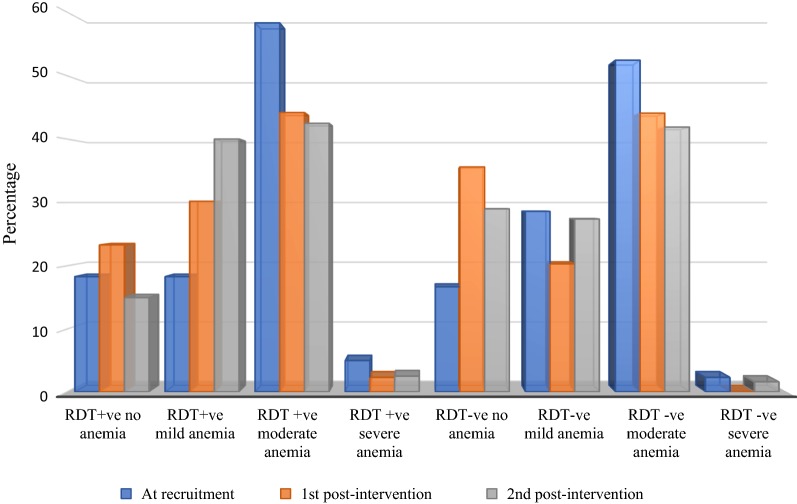
Anemia status relative to rapid diagnostic test results at recruitment, and during 1st and 2nd post intervention screenings. The proportion of RDT-positive children with moderate anemia was higher at recruitment (58.3%) than that at 2nd post-intervention screening (42.5%). The proportion of those with severe malaria was also higher at recruitment (5.0%) that at 2nd post-intervention screening (2.5%). The proportion of RDT-negative children with severe anemia at recruitment (2.3%) dropped to 0.0% at 1st and rose to 1.6% at 2nd post-intervention screenings

**Fig. 4:**
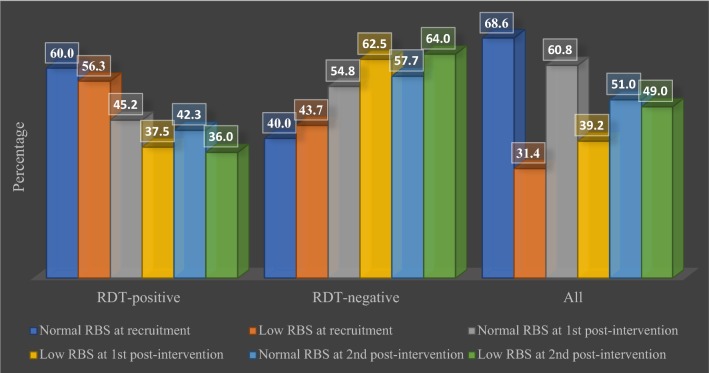
Relative proportion of children with normal or low random blood sugar (LRBS) level among RDT-positive and RDT-negative children. The proportion of RDT-negative children with normal random blood sugar steadily increased from 40.0% at recruitment, through 54.8% at 1st post-intervention screening and to 57.7% at 2nd post intervention screening, indicating that, probably, decrease in falciparum malaria parasitemia is associated with increase in random blood sugar

At recruitment into the study, there was a significant positive correlation between haemoglobin concentration and random blood sugar among RDT-positive (Pearson’s R = 0.26, P-value = 0.048) and RDT-negative (Pearson’s R = − 0.38, P-value = 0.02) children. After recruitment, there was no significant correlation between these two variables again.

There was a gradual decline in the mean *random blood sugar* (mg/dl) of all children at recruitment (94.8 ± 12.5), at 1st post-intervention screening (93.2 ± 15.2) and 2nd post intervention screening (90.3 ± 12.8). Though at recruitment, no significant difference in the mean RBS of RDT-positive and RDT-negative children was apparent, however, the mean RBS of RDT-positive males (n = 24, [53.3%]; 98.6 ± 12.0) was significantly higher (t = 1.88, P-value = 0.03) than that of RDT-positive females (n = 36 [63.2%]; 92.6 ± 12.3) and the mean RBS of RDT-negative males (n = 21 [46.7%]; 98.4 ± 10.6) was also significantly higher than that of RDT-negative females (n = 21 [36.8%]; 90.4 ± 13.5). Thus, overall, the mean RBS (mg/dl) of females (91.8 ± 12.7) was significantly lower (t = 2.83, P-value = 0.003) than that of males. A remarkable relationship was observed between gender and random blood sugar (r = − 0.268, P-value = 0.006). The mean RBS of all the children at recruitment (94.8 ± 12.5) was significantly higher (t = 2.54, P-value = 0.006) than that at the 2nd post-intervention screening (90.3 ± 12.8).

### Anaemia among children with or without malaria (Table 6)

The mean haemoglobin value (g/dl) at recruitment (9.6 ± 1.4) was significantly lower (t = − 3.17, P-value = 0.0009; t = − 2.664, P-value = 0.004, respectively) than the value at 1st post-intervention screening (10.2 ± 1.3) and 2nd post-intervention screening (10.1 ± 1.3). The prevalence of anaemia at recruitment and at first post-intervention measurement were 82.4% and 69.6% with Hb mean (± sd) of 11.6 (0.5) and 9.2 (1.2), respectively. At recruitment RDT-positive children were 2.16 times more likely to present with severe anaemia (χ^2^ = 0.02, P-value = 0.88, OR = 2.16, 95% CI 0.22, 21.49) and approximately 2 times more likely to present with moderate anaemia (χ^2^ = 0.17, P-value = 0.68, OR = 1.22, 95% CI 0.46, 3.22) in comparison to males. At 2nd post-intervention screening, the odds of RDT-positive children presenting with severe or moderate anaemia decreased from 2.16 and 2.06 to 1.31 and 1.02, respectively.

**Table 6:** Pre- and post-screening anemia status of RDT-positive and RDT-negative children before and after wearing pCAM

Anemia status	Malaria status	Hb at recruitment		Hb at 1st post-intervention measurement		t	P-value	Hb at 2nd post-intervention measurement		t	P-value
		Freq. (%)	Mean (± sd)	Freq. (%)	Mean (± sd)			Freq. (%)	Mean (± sd)		
All		102 (100.0)	9.6 (1.4)	102 (100.0)	10.2 (1.3)	− 3.17	0.0009	102 (100.0)	10.1 (1.3)	− 2.64	0.004
No anemia	Total	18 (17.7)	11.6 (0.5)	31 (30.4)	11.6 (0.6)	Not significant		24 (23.5)	11.8 (0.7)	Not significant	
	RDT +ve	11 (61.1)	11.5 (0.6)	10 (32.3)	11.4 (0.4)	Not significant		6 (25.0)	12.1 (0.9)	Not significant	
	RDT −ve	7 (38.9)	11.6 (0.4)	21 (67.7)	11.7 (0.6)	Not significant		18 (75.0)	11.7 (0.6)	Not significant	
With anemia	Total	84 (82.4)	9.2 (1.2)	71 (69.6)	9.5 (1.0)	− 1.67	0.048	78 (76.5)	9.6 (1.0)	− 2.30	0.011
	RDT +ve	49 (58.3)	9.0 (1.2)	33 (46.5)	9.4 (1.2)	Not significant		34 (43.6)	9.6 (1.2)	− 2.24	0.014
	RDT −ve	35 (41.7)	9.4 (1.1)	38 (53.5)	9.7 (0.8)	Not significant		44 (56.4)	9.6 (0.9)	Not significant	
Mild	All	23 (22.6)	10.4 (0.3)	25 (24.5)	10.4 (0.3)	Not significant		33 (32.4)	10.5 (0.3)	Not significant	
	RDT +ve	11 (47.8)	10.4 (0.4)	13 (52.0)	10.4 (0.2)	Not significant		16 (48.5)	10.4 (0.3)	Not significant	
	RDT −ve	12 (52.2)	10.5 (0.3)	12 (48.0)	10.4 (0.4)	Not significant		17 (51.5)	10.5 (0.3)	Not significant	
Moderate	All	57 (55.9)	8.8 (0.7)	45 (44.1)	9.1 (0.8)	− 2.02	0.02	43 (42.2)	9.1 (0.7)	− 2.12	0.02
	RDT +ve	35 (61.4)	8.8 (0.7)	19 (42.2)	8.8 (0.9)	Not significant		17 (39.5)	9.0 (1.0)	Not significant	
	RDT −ve	22 (38.6)	8.9 (0.8)	26 (57.8)	9.3 (0.7)	− 1.85	0.04	26 (60.5)	9.1 (0.6)	Not significant	
Severe	All	4 (3.9)	6.2 (0.6)	1 (1.0)	6.6 (0.0)	–		2 (2.0)	6.5 (0.1)	Not significant	
	RDT +ve	3 (75.0)	6.2 (0.8)	1 (100.0)	6.6 (0.0)	–		1 (50.0)	6.5 (0.0)	–	
	RDT −ve	1 (25.0)	6.2 (0.0)	0 (0.0)	0.0 (0.0)	–		1 (50.0)	6.4 (0.0)	–	

### Random blood sugar levels and anthropometry among children with or without malaria (Table 7 and Fig. 5)

When segregated by RDT status, there was no significant difference in the mean RBS of RDT-positive children at recruitment and at 1st and second post-intervention screenings. However, RBS of RDT-negative children was significantly higher (t = 1.86, P-value = 0.03) at recruitment (94.4 ± 12.6) than at 2nd post-intervention screening (89.9 ± 11.8).

**Table 7:** Pre- and post-screening random blood sugar levels among RDT-positive and RDT-negative children before and after wearing pCAM

Variable	Sub-variable	RBS at recruitment		RBS at 1st post-intervention measurement				RBS at 2nd post-intervention measurement			
		Freq. (%)	Mean (± sd)	Freq. (%)	Mean (± sd)	t	P-value	Freq. (%)	Mean (± sd)	t	P-value
Malaria status	All	102 (100.0)	94.8 (12.5)	102 (100.0)	93.2 (15.2)	Not significant		102 (100.0)	90.3 (12.8)	2.54	0.006
	RDT +ve	60 (58.8)	95.0 (12.4)	43 (42.2)	94.1 (18.6)	Not significant		40 (39.2)	91.0 (14.4)	Not significant	
	RDT −ve	42 (41.2)	94.4 (12.6)	59 (57.8)	92.4 (12.4)	Not significant		62 (60.8)	89.9 (11.8)	1.86	0.03
t-test (P-value)		Not significant		Not significant		–		Not significant		–

**Fig. 5:**
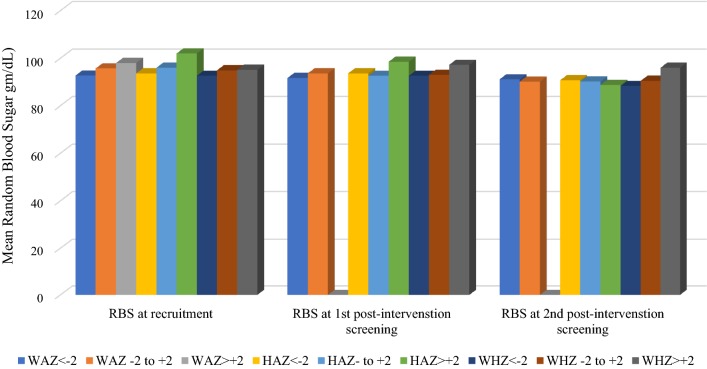
Mean random blood sugar (RBS) levels relative to nutritional anthropometry at recruitment, at 1st and at 2nd post-intervention screenings. At recruitment, malnourished children (WAZ < − 2), stunted children (HAZ < − 2) and wasted children (WHZ < − 2) had a relatively lower random blood sugar level than well-nourished (WAZ − 2 to + 2, HAZ − 2 to + 2 and WHZ − 2 to + 2) and obese (WAZ > + 2) children. Overall, there was a decline in random blood sugar throughout the study

Mean RBS was lowest among malnourished, stunted and wasted children at recruitment. Among children with normal WAZ (− 2 to + 2) mean RBS was significantly lower (t = 2.87, P-value = 0.002) at 2nd post-intervention (90.1 ± 11.9) than at recruitment (95.7 ± 12.0). Among those with normal HAZ (− 2 to + 2) mean RBS was also significantly lower (t = 2.48, P-value = 0.007) at 2nd post-intervention (90.2 ± 10.9) than at recruitment (96.0 ± 12.9). There was no significant difference in the mean RBS of children relative to their WHZ, at recruitment and at 1st and 2nd post-intervention screenings.

### Adverse effect monitoring

There was no reported skin allergy to pCAM at the end of the study. Throughout the study, there was no report of any allergic skin reaction among the study children or any of their siblings. Children with very low blood sugar were physically taken to the nearest primary or secondary health facility for management.

## Discussion

Malaria is not only endemic in metropolitan cities in Nigeria such as Lagos, it is also a disaster in its rural and coastal areas where low-income earners live in environment straddled with stagnant waters where mosquitoes easily breed, creating an ideal situation for the spread of malaria. According to Dewey and Meyer [20] and to Korpe and Petri [21], infections typically reduce the intake of nutrients and their bioavailability, increase nutrient and energy expenditure, and divert nutrients away from growth. This study was conducted within the first 4 months of the year during which food was expected to be adequate. The primary focus of this study was to evaluate whether “Moskeeto Armor^®^”, a protective clothing against malaria, influences malaria status of children aged under 5 years at two rural coastal communities in Lagos State. In addition, the study probed further into relations between malaria status, nutritional status, anaemia and hypoglycaemia condition. This study observed that about 60% of the study children were positive for RDT, indicating recent malaria infection. This was similar to the 55% prevalence earlier reported from a study in urban Lagos [22] but far higher than the 18% prevalence reported from another coastal community about 20 years ago [23]. Data gathered in this study suggests that the insecticide-treated protective clothing (“Moskeeto Armor^®^”) possibly protects children from mosquito bites before they sleep under insecticide-treated mosquito nets. At recruitment, the mean haemoglobin concentration of 9.6 g/dl indicated a pervasiveness of moderate anaemia among the study children. Anaemia prevalence of 82.4% observed in this study was higher than the 26.5% reported at another coastal community in Lagos [24], than the 64% reported from a study in Ghana [25], but similar to the more than 85% reported from Equatorial Guinea [26]. Anemia from sources such as malaria, haemoglobinopathies, nutritional deficiencies, iron-deficiency, helminthiasis, septicaemia or other childhood infections, is a major public health burden and a significant risk factor for child mortality, poor cognitive development and physical growth [27] as well as deprived immune mechanism [28].

The physiological factors responsible for increase in haemoglobin level of the children within the short period of study time is probably due to pCAM preventing mosquitoes to alight on and bite children wearing it before bed-time when they eventually sleep under LLINs. The pCAM gave these children further protection when they were supervised by their mothers/caregivers to wear the protective clothing and supplements the use of LLINs thus directly reducing malaria-induced anaemia morbidity and possible mortality. Malaria-induced anaemia can only occur when mosquitoes bite a child and malaria parasites invade, multiply within and eventually rupture the red blood cells.

This study also observed a relatively high prevalence of malnutrition (32.3%) and stunting (54.9%) among the study children, both occurring more in females. This contradicts the findings of Keino et al. [29] that stunting was more among boys than among girls, but agrees with the submission of Nigerian National Population Commission, ICF International and Nigeria Demographic and Health Survey (2013) that almost 30% of Nigerian children are underweight, more than double the proportion of neighbouring Ghanaian children who are underweight [30]. The almost 55% prevalence of stunting observed in this study was higher than the 47% reported from Central Africa [31], but lower than the 62% described in a study from Malawi [32]. Semba et al. also argued that stunting in children is associated with lower serum concentrations of all nine essential amino acids (tryptophan, isoleucine, leucine, valine, methionine, threonine, histidine, phenylalanine, lysine) of conditionally essential amino acids (arginine, glycine, glutamine), non-essential amino acids (asparagine, glutamate, serine), six different sphingolipids and with alterations in serum glycerophospholipid concentrations, compared with non-stunted children [32].

Korpe and Petri projected environmental enteropathy (EE) as having an important effect on stunting, a puzzling syndrome categorized by villus blunting, reduced intestinal epithelial surface area and absorptive capacity, altered gut mucosal barrier integrity, and immune-inflammatory changes [21]. Prendergast and Kelly proposed that stunting occurs in young children living in unsanitary settings [33] attesting to the condition where this study was conducted. According to the Sustainable Development Goals (SDGs), undernutrition is a major public health issue and its target 2.2 aspires to end hunger by 2030 [34]. Since under-fives are more at risk of malnutrition, it is therefore appropriate to use the nutritional status of the under-5-years population to draw conclusions about the situation of the whole population [35].

According to UNICEF, malnutrition contributed to 53% of deaths among under-fives in Nigeria, and levels of wasting and stunting are still very high [36]. The observed relatively high proportion of RDT-positivity among malnourished and stunted children, according to some studies, may indicate that resistance to infectious diseases and childhood anaemia are closely associated with the nutritional status of children [37, 38]. Mockenhaupt et al. [38] contended that malnutrition contributes to malaria-associated mortality. Malnutrition possibly postpones the normal development of the gut microbiota in early childhood or forcefully redirect it toward an altered composition that lacks the required functions for healthy growth and/or increases the risk for intestinal inflammation [39].

Though hypoglycaemia is a frequently encountered complication in falciparum malaria that is usually ascribed to increased glucose use and impaired glucose production caused by the inhibition of gluconeogenesis [40], it is not always documented in malaria studies among rural children in Africa. The 62.7% hypoglycaemia documented in this study was lower than the 47% reported elsewhere [41], though this study focused on severe malaria.

Why are all these variables related? First, this study further stresses the association between malaria and anaemia in under-fives which various studies have documented [42–45] extensively. Secondly, this study confirms the complex and inconclusive relationship effect of malnutrition on malaria [46]. There seems to be a vicious cycle between malaria and malnutrition as malaria imposes a heavier burden on malnourished children while malnutrition worsens malaria infection. An Ethiopian study resolved that contact with malaria parasites has a significant impact on the nutritional status of children [47]. The same study reported that in all probability, family income is a major determinant of whether or not children are malnourished which may exacerbate morbidity due to undernourishment in children living in malaria-endemic areas. Thirdly, malaria is associated with random blood sugar. Malaria and hypoglycaemia are clinically problematic and complex. A study reported that hypoglycaemia is a frequently encountered complication of falciparum malaria usually ascribed to increased glucose use and impaired glucose production caused by the inhibition of gluconeogenesis [48]. The pathophysiology of hypoglycaemia is beyond the scope of this paper, but it has been discussed and reported by Yau et al. [49], detailing a precise association and the pathways between malaria and hypoglycaemia. In this study, the proportion of children with malnutrition increased probably because of the worsening socio-economic factors which severely affected rural dwellers in the country. In addition, the proportion of RDT-negative children with normal random blood sugar (NRBS) increased from 40.0% at recruitment to 54.8% at 1st post-intervention screening and to 57.7% at 2nd post-intervention screening (Fig. 4) probably because there was a reduction in the biomass of falciparum malaria parasites, leading to increase in random blood sugar level as there was little or no competition between malaria parasites and the body cells for the available blood sugar.

Finally, there were mild differences between 1st and 2nd post intervention findings, especially in the haemoglobin concentrations. When mothers/caregivers were prompted to ensure that children wear the pCAM daily as from 17.00 h, the response was remarkable in the 1st month of study; the proportion of children with malaria and anaemia decreased significantly and the proportion of children with normal random blood sugar also increased significantly. However, in the 2nd month of study, when mothers/caregivers were not reminded to ensure children wear the pCAM, there was an apparent change in behaviour either due to memory lapse, forgetfulness or inability to perceive the wellness of the child lead to slight dip in haemoglobin concentrations. This highlights the importance of reminders in engaging mothers/caregivers in appropriate behavioural pattern towards the health of their children.

## Strengths and limitations

The study children and their parent/care-givers were community-based in a specific coastal region with little population dynamics. Materials for laboratory analysis were mostly supplied from USA ensuring no sub-standard item was used. Laboratory scientists for the study were professionals and well-trained. The study also employed the services of Social workers to visit community dwellers. Official letter of approval from the State to Local Government Authority facilitated recruitment of children. The study was carried out in the dry season. However, only RDT analysis was done. It would have been an advantage to have conducted thick and thin blood films for parasite density, speciation and stage of parasites as well as gametocytes. Analysis of sub-clinical malaria parasitemia using polymerase chain reaction (PCR) would have boosted the study. Certain factors may have affected the accuracy of the RBS test such as calibration of the Glucometer, ambient temperature, size of blood sample, high levels of certain substances (such as ascorbic acid) in blood, haematocrit, dirt on meter, humidity, and aging of test strips. In addition, this study did not measure baseline haemoglobinaemias or diabetes and these could be confounding factors.

Findings from this study may not necessarily be generalized to other children in Nigeria who are at risk of malaria, malnutrition, stunting or wasting. There are wide variations in life-style, socio-economic, demographic and environmental factors that determine these variables. Furthermore, the location of the study was the Atlantic coastline in southwest Nigeria, in contrast to what could have obtained in the arid northern Nigeria or the mountainous area in the middle of the country. Also, we did not study the deficiencies in central biogenic amines, relevant amino acids and micronutrients. A future study will determine these.

## Conclusion

This study has shown the effect of “Moskeeto Armor^®^”, a protective clothing worn against malaria, in two rural coastal communities on the Atlantic Ocean fringe of Lagos, Southwest Nigeria. In the 1st month of study only, but not in the 2nd month, social workers visited parents/care-givers daily, especially in the evening, to ensure that each child given the protective clothing wore it till child slept under insecticide-treated net. Social workers also gave some form of health education to the community. At recruitment, majority of the children tested positive for RDT while stunting and malnutrition were most prevalent, more among females than among males. Moderate anaemia was rampant among the children. At first post-intervention measurement, fewer children tested positive to RDT and there was improvement in the mean haemoglobin concentration of the children. There were more euglycaemic children who tested positive for RDT initially. Further studies are needed for a robust analysis of the correlation between malaria, malnutrition and hypoglycaemia. More efforts are needed not only the various tiers of the government but also by Partners and Donors to support elimination of child malnutrition in Nigeria towards achieving the Sustainable Development Goals.

### Recommendations

1. Decision-makers ought to start considering investing more healthcare funds in prevention programs.
2. Comprehensive longitudinal studies of the effects of protective clothing against mosquito bites and its effect on haemoglobin status of at-risk groups should be carried out.
3. The extent to which protective clothing supplements LLINs in well-nourished and undernourished children with anemia should be examined.
4. To articulate an effective control and prevention strategy, the association of malaria with anaemia, malnutrition with hypoglycaemia requires thorough investigation.

## Data Availability

All the data from this study are available from the Principal Investigator/Corresponding author.

## Abbreviations

ATP: Adenosine triphosphate
CI: Confidence interval
HAZ: Height-for-age Z-score
Hb: Haemoglobin
HIV: Human immunodeficient virus
LLINs: Long lasting insecticide-treated nets
LRBS: Low random blood sugar
NDHS: National Demographic and Health Survey
NHREC: National Health Research Ethics Committee
NRBS: Normal random blood sugar
OR: Odds ratio
pCAM: Protective clothing against mosquitoes
PCR: Polymerase chain reaction
RBS: Random blood sugar
RDT: Rapid diagnostic test
SD: Standard deviation
SDGs: Sustainable Development Goals
UNICEF: United Nations Children’s Fund
USA: United States of America
WAZ: Weight-for-age Z-score
WHO: World Health Organization
WHZ: Weight-for-height Z-score
